# The effect of traditional Chinese exercises on low Back pain and disability in middle-aged and older adults: A systematic review

**DOI:** 10.1016/j.pmedr.2025.103072

**Published:** 2025-04-14

**Authors:** Yanan Qi, Miaoqing Zhuang, Rui Liang, Shazlin Shaharudin

**Affiliations:** a Exercise & Sports Science Programme, School of Health Sciences, Universiti Sains Malaysia, Kubang Kerian, Kelantan, Malaysia; b Department of Nursing and Rehabilitation, Xi'an Jiaotong University City College, Xi'an, Shaanxi, China

**Keywords:** Exercise, Human health, Physical activity, Preventive medicine, Rehabilitation

## Abstract

**Objective:**

To assess the efficacy of traditional Chinese exercises (TCEs) in mitigating pain and disability among older and middle-aged individuals suffering from low back pain (LBP).

**Methods:**

From inception to November 2024, seven electronic databases were searched for TCEs treatment of LBP in the literature of randomized controlled trials: the Chinese Science and Technology Periodical Databases, PubMed, Embase, Cochrane Library, Web of Science, China National Knowledge Infrastructure, and Wanfang Data Knowledge Service Platform. The primary outcomes of emphasis were pain severity and disability. This review has been registered in the PROSPERO database (CRD42024626811). A meta-analysis was conducted using Review Manager 5.4 software.

**Results:**

Eleven of the 856 studies that were found through a search of seven electronic databases satisfied the requirements for inclusion. The conducted meta-analysis demonstrated a notable decrease in Visual Analogue Scale (VAS) pain scores [Mean Difference (MD) = −1.38, 95 % CI (−1.79, −0.97)] and Oswestry Disability Index (ODI) scores [MD = −4.36, 95 % CI (−6.84, −1.87)] for patients suffering from LBP who practiced TCEs, compared to those in the control group. The evidence for reductions in both VAS pain scores and ODI scores was rated as low quality in the Grading of Recommendations Assessment, Development and Evaluation.

**Conclusions:**

TCEs such as Tai Chi, Baduanjin, and Five-Animal exercises can help patients with LBP to some extent, but it is crucial to choose the appropriate type, intensity, and duration.

## Highlights

• Traditional Chinese Exercises can help reduce low back pain and improve function.
• Caution is needed for exercise type, intensity, and duration.
• Traditional Chinese Exercises encourage adherence to participate.

## 1 Introduction

Low back pain (LBP) has been identified as the second leading cause of disability worldwide, representing a significant public health concern (Maselli et al., 2019; Santos et al., 2020). Notably, LBP can occasionally manifest as a symptom of conditions not predominantly associated with musculoskeletal disorders, thereby complicating its diagnosis and treatment (Blödt et al., 2015; Bonakdar et al., 2019). LBP does not qualify as an independent diagnostic entity; rather, it is a multi-symptom condition. From a clinical perspective, it is typified by discomfort and dysfunction in the lumbar region, often concomitant with leg pain or neurological abnormalities in the lower extremities, and initially manifests as back pain (Dionne et al., 2008; Budhrani-Shani et al., 2016; Essman and Lin, 2022). The prevalence of LBP varies globally, affecting 58 % to 84 % of patients at some point in a lifetime (Wang et al., 2022). Furthermore, activity limitation due to LBP affects 7.3 % of the global population at any particular time, with a rising epidemiological trend observed in low- and middle-income countries (Enthoven et al., 2016). A noteworthy aspect is the recurrence of LBP, with approximately 33 % of cases reoccurring within a year following recovery from the previous episode (Essman and Lin, 2022; Buchbinder et al., 2018; Yang et al., 2012). LBP is currently the most common cause of disability and is a growing global burden on healthcare systems and society (Enthoven et al., 2016; Bontrup et al., 2019).

There has been some success with conventional therapies including medicine and surgery (Yuan et al., 2015; Jacobs et al., 2013). These therapies may not always work, though, and they can potentially have harmful side effects (Jacobs et al., 2013; Yang et al., 2013). As a result, many people have resorted to complementary and alternative medicine in search of more effective therapies (Furlan et al., 2012; Sherman et al., 2004). Similar opinions are expressed in a number of clinical guidelines regarding the management of LBP, which over the past 30 years has seen a greater focus on non-pharmacological therapies as opposed to pharmaceutical and surgical ones (Stochkendahl et al., 2018).

The low-intensity mind-body exercises, which emphasize posture coordination, breathing techniques, and meditation, include Tai Chi, Baduanjin, Yijinjing, Five-Animal Exercises, and Liuzijue (Huston and McFarlane, 2016; Cheng, 2015; Zhu et al., 2017; Ye and Jiang, 2021; Xiao et al., 2020). Traditional Chinese exercises (TCEs) are an old type of mind-body activity. Due to their affordability, safety, and ease of learning, TCEs have gained popularity in China as therapeutic activities for people with LBP (Qin et al., 2019; Hall et al., 2011). TCEs have a lot of benefits. First of all, they do not need costly equipment or specialist facilities, so they can be practiced whenever and wherever (Du et al., 2023). Secondly, TCEs efficiently increase muscle strength, balance, and aerobic capacity (Xie et al., 2024; Qiu et al., 2024; Zheng et al., 2019; Wang et al., 2023). Thirdly, TCEs have significant clinical utility because of their inexpensive cost and straightforward requirements, which enable smooth community adoption (Lan et al., 2013; Guo et al., 2016).

A substantial body of research has been dedicated to conventional therapeutic modalities such as pharmacotherapy and surgical interventions for the management of LBP. However, these conventional approaches often fail to produce optimal outcomes and may entail potential adverse effects (Jacobs et al., 2013; Yang et al., 2013). Consequently, there is a growing interest in complementary and alternative medicine options, particularly non-pharmacological interventions such as TCEs (Furlan et al., 2012; Sherman et al., 2004). Nevertheless, there is a paucity of comprehensive evidence regarding the efficacy of TCEs specifically among middle-aged and older adults. This systematic review aims to address this gap by critically assessing the impact of TCEs on pain and disability in this demographic, providing empirical validation and theoretical support for therapeutic approaches to LBP.

## 2 Methods

The Preferred Reporting Items for Systematic Reviews and Meta-Analyses standards have been strictly followed in this systematic review (Tugwell and Tovey, 2021). This review has been registered in the PROSPERO database (CRD42024626811) (Schiavo, 2019).

### 2.1 Search strategy

To find full-text publications of relevant studies, we performed comprehensive searches across seven electronic databases: the Chinese Science and Technology Periodical Databases, PubMed, Embase, Cochrane Library, Web of Science, China National Knowledge Infrastructure, and Wanfang Data Knowledge Service Platform. Literature searches in each database were from inception to November 2024. The following Medical Subject Headings search phrases were used to restrict the search to human research and publications in both Chinese and English languages. The following search terms were utilized: (“Traditional Chinese Exercises” OR “Tai Ji” OR “Tai-ji” OR “Tai Chi” OR “Chi, Tai” OR “Tai Chi Chuan” OR “Taiji” OR “Taijiquan” OR “T'ai Chi” OR “Tai Ji Quan” OR “Ji Quan, Tai” OR “Quan, Tai Ji” OR “Baduanjin” OR “Yijinjing” OR “Qigong” OR “Liuzijue” OR “Wuqinxi” OR “Five-animal exercises”) and (“Low Back Pain” OR “Back Pain, Low” OR “Back Pains, Low” OR “Low Back Pains” OR “Pain, Low Back” OR “Pains, Low Back” OR “Low Back Ache” OR “Ache, Low Back” OR “Aches, Low Back” OR “Back Ache, Low” OR “Back Aches, Low” OR “Low Back Aches” OR “Low Backache” OR “Backache, Low” OR “Backaches, Low” OR “Low Backaches” OR “Lower Back Pain” OR “Back Pain, Lower” OR “Back Pains, Lower” OR “Lower Back Pains” OR “Pain, Lower Back” OR “Pains, Lower Back” OR “Lumbago” OR “Low Back Pain, Mechanical” OR “Mechanical Low Back Pain” OR “Low Back Pain, Posterior Compartment” OR “Low Back Pain, Postural” OR “Postural Low Back Pain” OR “Low Back Pain, Recurrent” OR “Recurrent Low Back Pain”). A comprehensive search of the bibliographic references of pertinent reviews yielded additional publications. Additionally, a thorough examination was conducted of the World Health Organization International Clinical Trials Registry Platform and the China Clinical Trials Registry, with the objective of identifying ongoing or unpublished trials. In instances where further clarification was required, the reviewers contacted the authors of the study.

Inclusion criteria:

(1) Study type: We only included published reports of randomized controlled trials examining how TCEs affect LBP. Only human studies in English and Chinese publications were allowed.
(2) Type of participants: at least one group of participants with a minimum mean age of 40 years and a clinical diagnosis of LBP. Nationality and gender were unrestricted.
(3) Interventions: A range of TCEs, including Liuzijue, Tai Chi, Baduanjin, Yijinjing, Qigong, and Five-Animal Exercises, were implemented in the intervention. The clinical trials encompassed a range of interventions, including TCEs in comparison with a control group that did not receive any intervention, a group that received a placebo (a waiting list with no alterations to lifestyle), and alternative therapies such as physical activity, massage, and exercise therapy.
(4) Types of outcome measures: At least one of the two assessments (pain and disability) discussed below should be included in the outcome measures.

Studies that satisfied one of the following criteria were disqualified:

(1) trials that are not randomized; (2) conference proceedings or abstracts; (3) recurrent publications; (4) incomplete or missing data; (5) editorials, reviews, opinion pieces, and systematic and narrative review articles; (6) case series and case studies; (7) research without a control group; (8) interventions that combined TCEs with other exercises. Differences in assessment were discussed until consensus was reached.

Data extraction:

Using criteria for inclusion and exclusion depending on the population, intervention, control, comparator, and outcome, QY and ZM conducted an initial screening of article titles and abstracts at the conclusion of the search (Table 1). The name of the author, year of publication, sample size, participants' average age, specific type of exercise, duration of the intervention, specifics of the intervention program, observation indicators, and primary results were then extracted by QY from each included study.

**Table 1:** Population, Intervention, Control/Comparator, and Outcome.

Population	Middle-aged and older adults
Intervention	Traditional Chinese Exercises
Control/comparator	Other forms of intervention or no intervention
Outcome	Pain · Visual Analog Scale · Short Form McGill Pain Questionnaire Disability · Modified Oswestry Lumbar Dysfunction Index · Roland-Morris Questionnaire Other measures · Mechanical performance indicators of low back muscle groups peak torque, peak torque to body weight ratio, average power, total work, transversus abdominis flexion/extension ratio, electromyographic signal · Sleep diary, Pittsburgh Sleep Quality Index · Intra-abdominal pressure test · Lower extremity neuromuscular function indicators · Knee and Hip Position Sense · Depression Scale · Self-efficacy · Hand grip strength test

### 2.2 Risk of bias

The methodological quality of the selected studies was evaluated by QY and ZM using the Cochrane Risk of Bias evaluation tool (Review Manager 5.4), and LR was responsible for resolving any disagreements that arose. The evaluation was predicated on a number of biases, including insufficient outcome data, selective reporting, participant and staff blinding, allocation confirmation, random sequence creation, and outcome assessment blinding. As a result, each included study was divided into three categories: (1) unclear risk (i.e., insufficient evidence), (2) low risk, and (3) high risk.

### 2.3 Grading of recommendations assessment, development and evaluation (GRADE) evidence grading evaluation

In this study, the quality of the evidence was rigorously assessed using the GRADE profiler 3.6, a sophisticated tool designed for grading the quality of evidence and strength of recommendations. The evidence was systematically categorized into four echelons: high, moderate, low, and very low quality. This comprehensive evaluation process encompassed an in-depth analysis across five critical domains: risk of bias, inconsistency, indirectness, imprecision, and publication bias. Each domain was meticulously scrutinized to ascertain the robustness and reliability of the evidence under consideration.

### 2.4 Statistical methods

A comprehensive meta-analysis was performed, employing the standardized mean difference (MD) alongside 95 % CI as the key effect size metrics for continuous data. The heterogeneity among the included studies was assessed using the Chi-squared test and I-squared (*I*^2^) statistic. The selection of the analytical model was contingent on the observed level of heterogeneity: in instances where the *I*^2^ value indicated less than 50 % heterogeneity and the *P* value exceeded 0.1, indicating homogeneity across studies, a fixed-effects model was employed. Conversely, a random-effects model was employed to account for greater variability. Subgroup analyses were conducted to explore sources of heterogeneity and evaluate the robustness of the results, supplemented by sensitivity analyses. These sensitivity analyses involved methodological transformations of the analytical models and sequential exclusion of individual studies to assess their impact on the overall findings. This rigorous approach ensures a thorough examination of the data, enhancing the reliability of the conclusions. All statistical analyses were conducted using Review Manager 5.4.

### 2.5 Ethics approval and consent to participate

As this is a systematic review, the acquisition of data from human subjects was not necessary. Therefore, ethical approval was not required. Given the nature of this study, written informed consent was deemed unnecessary.

## 3 Results

### 3.1 Selection of studies

**Fig. 1:**
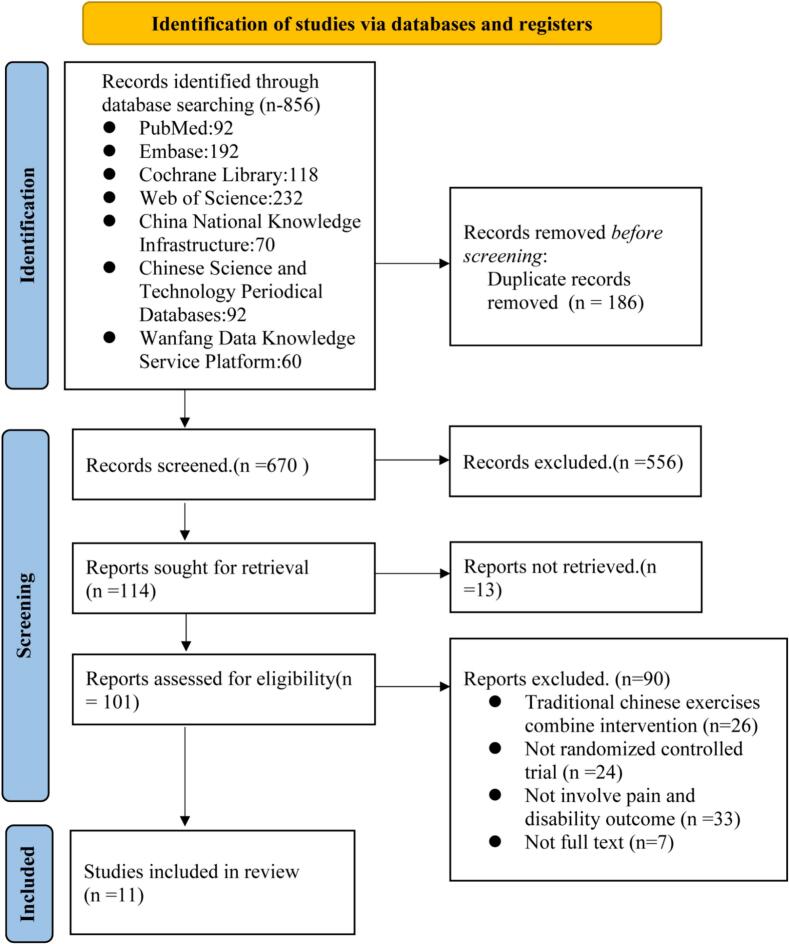
Preferred Reporting Items for Systematic Reviews and Meta-Analyses flowchart of the trial screening process.

A total of 856 items were identified in the initial search (Fig. 1). Subsequent to the elimination of 186 duplicate entries, the total number of articles was reduced to 670. After a thorough review of the abstracts and titles of the remaining articles, 556 articles were excluded. Ultimately, 11 studies were deemed suitable for this review.

### 3.2 Research quality assessment

**Fig. 2:**
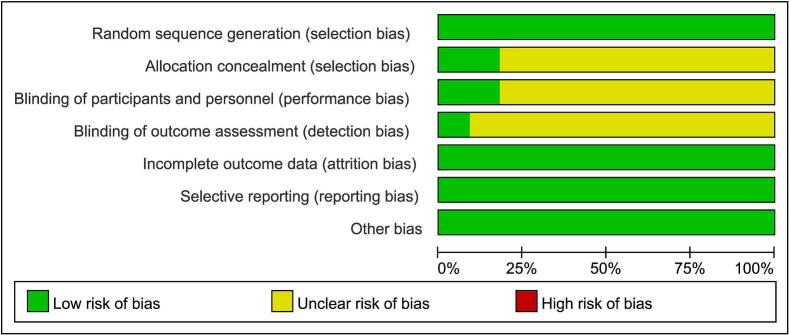
Risk of Bias in Randomized Controlled Trials Examining How Traditional Chinese Exercises Affect Low Back Pain in Adults Over 40 Years of Age, Database Search from Inception to 2024.

The Cochrane risk of bias was employed to ascertain the inclusion of randomized controlled trials of varying quality in this systematic review (Fig. 2). This figure shows the risk of bias assessment of each study from the systematic review, which typically showed good study quality. The majority of studies showed good performance in handling inadequate outcome data, selective reporting, and randomized sequence generation, all of which were deemed to be low risk. Nonetheless, a few high-risk instances of allocation concealment and outcome assessor blinding were observed, indicating the need for additional enhancements to guarantee experimental design rigor and data collecting impartiality. Nevertheless, most of the trials did relatively well in controlling for various types of bias, but there is a need to focus more on specific areas in the future and to continually monitor for new information to address new challenges that may arise.

### 3.3 Characteristics of participants

The total sample size of all the included research comprised 766 people. There were 20 individuals in the smallest sample size and 120 participants in the biggest. One study included only women, while two studies included only men (Yan et al., 2022; Sun et al., 2021; Wang et al., 2017). Participants in the remaining eight trials ^43–50^ comprised both male and female. In addition, all participants in the 11 studies were 50 years of age or older and had been diagnosed with chronic LBP (Li et al., 2022; Fang et al., 2015; Chen et al., 2023; Ding and Wang, 2014; Liu et al., 2021; Zou et al., 2019; Teut et al., 2016; Liu et al., 2019).

### 3.4 Characteristics of the interventions in the control and experimental groups

The following were the interventions given to the control group. Non-intervention: Without further interventions, the control group continued their regular daily routine and activities. Exercise intervention: the control group received rehabilitation exercises and a core muscle training program. Physical therapy interventions: the control group received magnetic heat therapy and interferential electrical therapy. Other interventions: medication, lifestyle optimization, routine health education, aerobic exercise, and a two-by-two approach. In order to thoroughly assess the efficacy of the various intervention techniques, the control group interventions were generally multidimensional.

The interventions in the experimental group consisted mainly of four types of TCEs: Tai Chi, Baduanjin, Five-animal exercises and Qigong. Tai Chi exercises alone were performed in four studies: water Tai Chi, traditional Tai Chi, Chens Tai Chi, and modified Chens Tai Chi (Yan et al., 2022; Chen et al., 2023; Zou et al., 2019; Liu et al., 2019). There was only one study that performed Baduanjin exercises alone for vertical Baduanjin training (Ding and Wang, 2014). There was only one study that performed Five-animal exercises alone (Fang et al., 2015). There was also only one study of Qigong exercises alone (Teut et al., 2016).

There were four studies that added TCEs to the control group, and all of them added Baduanjin exercises to the control group (Sun et al., 2021; Wang et al., 2017; Li et al., 2022; Liu et al., 2021). In summary, the interventions in the experimental group were based on TCEs alone, including various forms of Tai Chi, Baduanjin, Five-animal exercises, and Qigong, and there were also some studies that added the TCEs Baduanjin to the control group for the intervention. Six weeks was the shortest intervention time, whereas 24 weeks was the longest. Furthermore, the frequency of exercise varied from three to five sessions per week. Sessions lasted anything from 15 min to 90 min on average. The average length of a session was 30 to 60 min. Table 2 provides a summary of the characteristics of the included studies.

**Table 2:** Characteristics Summary of Randomized Controlled Trials Examining How Traditional Chinese Exercises Affect Low Back Pain in Adults Over 40 Years of Age, Database Search from Inception to 2024.

Author	Sample size	Mean age	Type	Duration	Intervention		Observation indicators
					Experimental group intervention	Control group intervention	
Sun et al., 2021	*N* = 70 Control group = 25 Experimental group = 18 Dropout = 27	Control Group = 71.44 ± 4.28 Experimental group = 70.71 ± 3.89	Seated Baduanjin	24 Weeks	Control group modality + seated Baduanjin (single exercise duration within 15 min, adhere to every other day regular morning and evening exercise once, ensure exercise more than 20 times in 4 weeks, continuous intervention for 6 months)	Optimize lifestyle interventions + medications	VAS^1^, Modified ODI^2^, Bone Mineral Density
Li et al., 2022	*N* = 80 Control group = 39 Experimental Group = 37 Dropout = 4	Control Group = 70.74 + 6.96 Experimental group = 68.86 + 4.63	Baduanjin	12 weeks	Control group modality + Baduanjin (45 min/d, 4 d/week, 12 weeks)	Routine health education for chronic low back pain	VAS, Modified ODI
Fang et al., 2015	*N* = 72 Control group = 31 Experimental Group =32 Dropout = 9	Control Group = 53.88 ± 14.17 Experimental group = 52.91 ± 15.80	Five-animal exercises	24 weeks	Five-animal exercises Program (Patients can generally practice the whole set, or choose to practice 1–2 of the pieces. The exercise intensity is controlled at 60 % ∼ 70 % of the maximum heart rate. The time of each exercise is controlled at 30–60 min, 3–4 times a week for 6 months).	Rehabilitation Gymnastics (using the rehabilitation gymnastics program from McKenzie Gymnastics Au, with each session lasting 30–60 min, 3–4 times a week for 6 months)	Short Form McGill Pain Questionnaire, lumbar and abdominal muscle group mechanical performance indicator
Chen et al., 2023	*N* = 74 Control group = 37 Experimental group = 37	Control Group = 68. 18 ± 8.79 Experimental group = 67.89 ± 8.57	water Tai Chi	8 weeks	Water Tai Chi Training (30 min, including 5 min each for warm-up and relaxation. 3 times/w. 8 *w*/w consecutive, 8 w for 1 class)	Core muscle training program (30 min/times. 3 times/w, continuous training for 8 w, 8 w for 1 treatment course)	Short Form McGill Pain Questionnaire, sleep diary, Pittsburgh Sleep Quality Index
Ding and Wang, 2014	*N* = 40 Control Group = 18 Experimental group = 22	Control Group = 60.89 + 4.92 Experimental group = 61.05 + 4.66	Vertical Baduanjin	12 Weeks	Vertical Baduanjin Training (5 reps/w, 40 min/rep, workout time is 12 w)	Take ibuprofen extended-release capsules for pain	VAS, modified ODI, lumbar lordosis, sacral tilt angle, lumbar mobility
Wang et al., 2017	*N* = 119 Control Group = 45 Experimental group = 45 Dropout = 29	Control Group = 66.55 + 3.55 Experimental group = 66.72 + 3.21	Baduanjin	12 weeks	Control group modality + Baduanjin (2–3 sessions of Baduanjin exercise each early in the morning and after dinner, after a moderate amount of food and drink, and after emptying urine and feces)	Magnetic heat treatment + interferential electric therapy (the treatment time is 20 min, once a day, 3–4 times a week, 4 weeks for a course of treatment, a total of 3 courses)	VAS, Roland-Morris Questionnaire, intra-abdominal pressure test
Liu et al., 2021	*N* = 120 Control group = 53 Experimental Group = 47 Dropout = 20	Control Group = 60.92 + 7.52 Experimental group = 58.34 + 4.89	Baduanjin	12 Weeks	Control group: Treatment + Baduanjin (practiced five times a week for at least 30 min each time). (See the 2003 edition of the State General Administration of Sport for the specific movements of Baduanjin).	The control group was guided by professionals for recovery treatment (included medication and aerobic exercise. Aerobic exercise includes walking, jogging, cycling)	VAS, modified ODI
Zou et al., 2019	*N* = 28 Control group = 13 Experimental group = 15	Control Group = 60.92 + 7.52 Experimental group = 60.67 + 2.58	Tai Chi	12 weeks	Modified Chens Tai Chi movements (60 min per session, three times a week for 12 weeks)	Control Group Maintains Normal Daily Activities	VAS, lower extremity neuromuscular function indicators
Yan et al., 2022	*N* = 20 Control group = 10 Experimental group = 10	Control Group = 70.00 + 1.26 Experimental group = 68.00 + 1.15	Tai Chi	6 weeks	Tai Chi (practice Tai Chi 3 times a week for 6 weeks. Each session lasts 60 min)	Control Group Maintains Normal Daily Activities	VAS, spatio-temporal gait measures, dynamic balance measures
Teut et al., 2016	*N* = 115 Control Group = 57 Experimental group = 58	Control Group = 72.6 ± 6.0 Experimental group = 72.4 ± 5.7	Qigong	12 Weeks	Qigong Group (12 sessions of 90 min for 3 months)	Control group receives no additional intervention	VAS, Short Form −36, depression scale, self-efficacy, grip strength test
Liu et al., 2019	*N* = 28 Control Group = 13 Experimental group = 15	Control Group = 60.67 ± 2.58 Experimental group = 58.13 ± 5.38	Chens Tai Chi	12 Weeks	Chens Tai Chi Chuan (60 min three times a week for 12 weeks)	Control group receives no additional intervention	VAS, knee and ankle joint position sense

1. VAS: Visual Analogue Scale.

2. ODI: Oswestry Disability Index.

### 3.5 Summary of Main outcomes included in the studies

This study synthesized the findings of eleven studies to assess the effectiveness of TCES in relieving pain and dysfunction in patients with LBP (Table 3). Among the studies, nine of the studies employed the Visual Analog Scale (VAS) to assess pain levels, while two studies utilized the Short Form McGill Pain Questionnaire to evaluate the difference in pain between the two groups. The findings of nine of the eleven studies indicated that TCEs were effective in reducing pain; however, two studies yielded contradictory results. Additionally, four studies utilized the Oswestry Disability Index (ODI) or Roland-Morris Questionnaire to evaluate lumbar spine dysfunction, with all demonstrating that TCEs significantly improved the function of the lumbar spine in patients. Secondary outcome indicators, including peak torque, average power, electromyographic signal, and sleep quality, demonstrated that TCEs not only improved lumbar spine function in patients, but also improved sleep quality, spinal mobility, and gait to a certain extent. However, they had no significant effect on knee and hip positional sense and intra-abdominal pressure.

**Table 3:** Main Outcomes Summary From Randomized Controlled Trials Examining How Traditional Chinese Exercises Affect Low Back Pain in Adults Over 40 Years of Age, Database Search from Inception to 2024.

Author	Sample size	Duration	Main outcomes		Experimental group (Mean ± SD)		Control group (Mean ± SD)		*p*-value
					pre	post	pre	post	
Sun et al., 2021	N = 70	24 Weeks	VAS^1^ (cm)		5.18 ± 1.24	1.22 ± 0.92	4.99 ± 1.47	1.96 ± 1.03	0.02
			ODI^2^		21.45 ± 5.30	10.83 ± 2.64	23.08 ± 5.01	12.67 ± 2.79	0.03
Li et al., 2022	N = 80	12 weeks	VAS (mm)		49.19 ± 5.94	30.51 ± 7.42	48.18 ± 6.40	36.59 ± 9.77	P<0.01
			ODI		14.62 ± 2.81	10.38 ± 3.16	15.10 ± 4.26	14.00 ± 6.39	P<0.01
Fang et al., 2015	N = 72	24 weeks	SF-MPQ^3^	PRI^4^	14.32 ± 6.03	1.31 ± 1.73	14.36 ± 6.54	2.86 ± 3.52	P<0.05
				VAS (cm)	5.22 ± 1.29	1.25 ± 1.34	4.95 ± 1.93	2.05 ± 1.92	P<0.05
				PPI^5^	2.74 ± 0.93	0.67 ± 0.61	2.80 ± 1.06	1.14 ± 1.07	P<0.05
				Composite Score	22.28 ± 7.13	3.19 ± 3.29	22.12 ± 8.16	6.05 ± 6.09	P<0.05
Chen et al., 2023	N = 74	8 weeks	SF-MPQ	PRI	12.25 ± 2.30	6.18 ± 1.59	12.46 ± 2.25	8.21 ± 3.57	P<0.05
				VAS (cm)	6.87 ± 1.92	2.58 ± 0.42	6.83 ± 1.83	3.57 ± 0.02	P<0.05
				PPI	3.80 ± 1.24	1.27 ± 0.35	3.79 ± 1.33	1.68 ± 0.37	P<0.05
Ding and Wang, 2014	N = 40	12 Weeks	VAS (cm)		5.56 ± 1.13	2.90 ± 0.64	5.36 ± 1.11	3.94 ± 0.86	P<0.01
			ODI		32.27 ± 2.67	22.14 ± 5.39	31.61 ± 4.27	27.06 ± 3.93	P<0.01
Wang et al., 2017	N = 119	12 weeks	VAS (cm)		6.45 ± 1.05	1.66 ± 0.57	6.48 ± 0.94	3.06 ± 0.88	P<0.01
			Roland-Morris Questionnaire		13.90 ± 2.17	4.33 ± 0.57	14.10 ± 2.04	6.46 ± 1.40	P<0.01
Liu et al., 2021	N = 120	12 Weeks	VAS (cm)		6.06 ± 1.13	2.46 ± 1.45	6.34 ± 1.16	3.77 ± 1.33	P<0.01
			ODI		27.38 ± 6.97	17.20 ± 7.73	28.40 ± 7.66	25.00 ± 6.94	P<0.01
Zou et al., 2019	N = 28	12 weeks	VAS (cm)		5.67 ± 0.81	3.47 ± 0.99	5.85 ± 0.89	5.85 ± 0.8	P<0.01
Yan et al., 2022	N = 20	6 weeks	VAS (cm)		5.50 ± 1.35	4.40 ± 0.97	5.40 ± 1.17	5.50 ± 1.08	P<0.05
Teut et al., 2016	N = 115	12 Weeks	VAS (mm)		50.6 ± 19.5	34.14 (95 % CI:28.51–39.78)	50.6 ± 21.3	41.25 (95 % CI:36.07–46.42)	*P* = 0.06
Liu et al., 2019	N = 28	12 Weeks	VAS (cm)		5.67 ± 0.81	3.47 ± 0.99	5.85 ± 0.89	5.85 ± 0.8	P<0.01

*P*-values were determined by the authors of the original studies, who employed a range of statistical methodologies to do so. These methods include, but are not limited to, *t*-tests and multiple linear regression analysis, among others.

1. VAS: Visual Analogue Scale.

2. ODI: Oswestry Disability Index.

3. SF-MPQ: Short Form McGill Pain Questionnaire.

4. RPI:Pain Rating Index.

5. PPI:Present Pain Intensity.

### 3.6 Meta-analysis results

#### 3.6.1 Vas

**Fig. 3:**
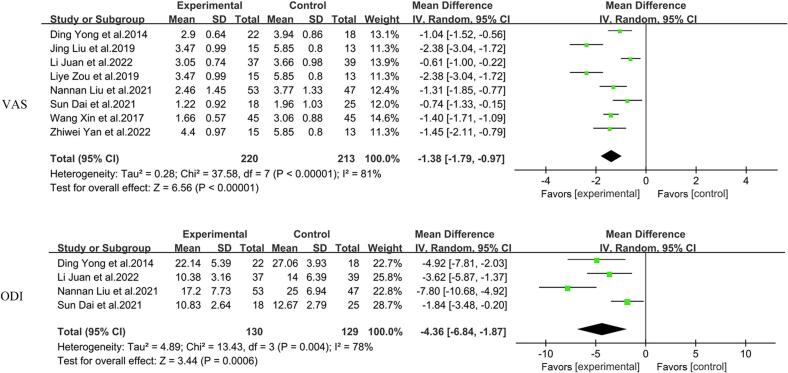
Meta-analytic Results of the Effect of Traditional Chinese Exercises on Visual Analogue Scale and Oswestry Disability Index for Low Back Pain.1.VAS: Visual analog scale.2.ODI: Oswestry disability index.3.Mean difference (MD): The difference in means between the experimental group and the control group.4.IV. Random, 95 % CI: Inverse Variance. Mean difference with a 95 % confidence interval using a random-effects model.5.Heterogeneity: Measures of heterogeneity among studies, including Tau^2^, Chi^2^, degrees of freedom (df), and *I*^*2*^.6.Test for overall effect: *Z*-test and its corresponding *P*-value to assess the overall effect.

Among the studies, nine studies used the VAS tool to assess post-intervention pain, of which the outcome data in Teut et al., (2016) study showed only 95 % CI and were not included in the study of VAS heterogeneity, while the remaining eight studies had heterogeneity among themselves (*I*^2^ = 81 %, *P* < 0. 01), and using a random effects model, the results showed that the VAS scores of the experimental group were lower than those of the control group [MD = −1.38, 95 % CI (−1.79, −0.97)]. Heterogeneity was still significant after sensitivity analysis, and subgroup analysis was performed for duration of intervention as well as type of intervention, which showed that heterogeneity was reduced after subgroup analysis by duration of intervention (*I*^2^ = 80.8 %, P < 0. 01) and type of intervention (*I*^2^ = 50.2 %, *P* < 0.05), and all differences between groups were statistically significant (P < 0.05) (Fig. 3).

#### 3.6.2 ODI

Among the studies, four studies used the ODI to assess low back disability after intervention, and the results showed heterogeneity among studies (*I*^2^ = 78 %, P < 0. 01), and using a random effects model, the results showed that the ODI scores of the experimental group were lower than those of the control group [MD = -4.36, 95 % CI (−6.84, −1.87)]. Heterogeneity was still significant after sensitivity analysis, and subgroup analysis was performed for intervention duration, which showed that heterogeneity decreased after subgroup analysis by intervention duration (*I*^2^ = 61 %, *P* = 0.08) (Fig. 3).

### 3.7 Sensitivity analysis

In the sensitivity analysis, by transforming the model and excluding studies one by one, it was observed that the effect sizes of the individual studies did not change significantly. This suggests that the results are relatively robust. Specifically, this review employed a rigorous sensitivity analysis methodology that included adjustments to the model specifications and sequential exclusion of individual studies to examine the consistency and stability of the overall findings. The analyses showed that even when model parameters were changed or specific studies were excluded, the effect sizes remained largely consistent with no notable fluctuations. These observations support the reliability and robustness of the research conclusions.

### 3.8 GRADE evidence quality evaluation

The quality of the evidence for each outcome measure incorporated into the meta-analysis was evaluated using the GRADE approach. This assessment revealed that the evidence supporting reductions in both VAS and ODI was deemed to be of low quality. This grading underscores the need for additional high-quality studies to strengthen confidence in these findings.

## 4 Discussion

### 4.1 Summary of Main findings

The findings suggest that TCEs such as Qigong, Tai Chi, Baduanjin, and Five-Animal exercises have positive effects on relieving pain and dysfunction in patients with LBP. However, no significant differences were observed in some of the studies, which may be related to the shorter duration of a single practice session and the insufficient number of sessions. For instance, in a study (Sun et al., 2021), the experimental group engaged in 15 min of Baduanjin exercises each session, while in another study (Teut et al., 2016) identified that the schedule of only 12 sessions over three months could be a contributing factor to the non-significant outcomes. These findings imply that the duration and frequency of practice should be considered when designing an intervention program for TCEs to optimize treatment outcomes. While the majority of studies support the effectiveness of TCEs, the available evidence is predominantly based on small sample sizes and brief intervention periods. Consequently, there is a pressing need for high-quality, large-scale studies in the future. These studies should investigate the mechanism of action of TCEs and their long-term efficacy. Additionally, they should explore the possibility of combining TCEs with other therapeutic approaches. This would provide patients with LBP more personalized and efficient treatment options.

### 4.2 Grading of quality of evidence

The quality of the included literature was generally high, with a low risk of bias; however, most studies did not mention the use of assessor blinding in outcome measurement. Future research should aim to improve the design related to blinding. In the GRADE assessment, the evidence levels for both VAS and ODI outcomes were rated as low, and even after conducting sensitivity analyses, heterogeneity persisted across studies. The risk of bias for all outcome measures was downgraded due to issues with allocation concealment and inadequate implementation of blinding methods. Consequently, researchers should prioritize standardizing experimental designs and meticulously executing these processes in future studies.

### 4.3 Analgesic mechanisms of TCEs in LBP

The existing body of research on the effects of TCEs on LBP is currently limited. However, several studies have suggested that exercise can provide direct pain relief by promoting the release of substances such as endorphins (Fuentes et al., 2011). Furthermore, exercise may enhance pain management through the placebo effect, particularly when individuals hold positive expectations about its analgesic effects (Rossettini et al., 2018; Rossettini et al., 2023; Ezzatvar et al., 2024). The present study demonstrated that TCEs exhibited superior efficacy in alleviating pain and disability in patients with LBP, in comparison to a control group that did not undergo any intervention. These findings imply that TCEs exert a positive influence on the body at the physical level and further enhance their analgesic effects by modulating the neuroendocrine system to promote the release of endogenous analgesic substances. However, to comprehensively elucidate the mechanism of action of TCEs, further exploration is necessary to investigate their specific pathways of action at the physiological and psychological levels, and to verify their long-term efficacy and safety. Concomitantly, given the influence of contextual and placebo effects on motor behavior and pain perception, future studies should prioritize the optimization of these factors to enhance therapeutic effects.

### 4.4 Clinical efficacy of TCEs in LBP

The clinical efficacy of TCEs for LBP warrants further investigation and exploration. Existing studies indicate that TCEs show significant benefits in alleviating pain and improving disability among patients with LBP, yet there is notable heterogeneity in the results. Subgroup analyses reveal that this heterogeneity primarily stems from factors such as the duration of intervention, types of interventions, and possibly the duration of individual sessions. The absence of standardized guidelines for the frequency and duration of TCEs in the treatment of LBP underscores the necessity for future research to explore these variables. The development of more scientific and standardized application guidelines is imperative to enhance the accuracy and effectiveness of TCEs in clinical practice.

## 5 Strengths and limitations

There are certain restrictions on this review. Firstly, the trustworthiness of the study findings may have been somewhat diminished by the modest number of studies on the two end measures of LBP pain and disability. Secondly, there were differences in the intervention techniques used in the included research, which mostly included four types: Qigong, Tai Chi, Baduanjin, and Five-Animal exercises. As a result, the outcomes were somewhat more heterogeneous. Furthermore, there were differences in the length of each exercise, the frequency of the intervention, and the duration of the intervention throughout the included trials. These factors contribute somewhat to the heterogeneity of the results. The theoretical foundation for treating middle-aged and older patients with LBP is provided by this study, which not only promotes an economical and successful approach to treating LBP but also conserves national health resources and lessens the financial strain on patients. Future research should identify the ideal exercise frequency and intervention time as well as further standardize the TCEs pattern.

## 6 Conclusion

In conclusion, this series of studies suggests that incorporating TCEs into a program for the management of LBP is a feasible and effective strategy, particularly for relieving pain symptoms and improving quality of life. However, to optimize its efficacy, it is essential to customize factors such as the type, intensity, and duration of exercise, taking into account individual circumstances.

## CRediT authorship contribution statement

**Yanan Qi:** Writing – review & editing, Writing – original draft, Methodology, Formal analysis, Conceptualization. **Miaoqing Zhuang:** Writing – original draft, Methodology, Data curation. **Rui Liang:** Writing – original draft, Methodology. **Shazlin Shaharudin:** Writing – review & editing, Supervision, Resources, Project administration.

## Funding

The study was funded by Ministry of Higher Education Malaysia Fundamental Research Grant Scheme with Project No: R503-KR-FRG001-0000000694-K134.

## Declaration of competing interest

The authors declare that they have no known competing financial interests or personal relationships that could have appeared to influence the work reported in this paper.

## Figures and Tables

**Table:** 

Database	Search String
Pubmed	Search: (((((((((((((((((((traditional Chinese exercises[Title/Abstract]) OR (Tai Ji[Title/Abstract]))) OR (Tai-ji[Title/Abstract])) OR (Tai Chi[Title/Abstract])) OR (Chi, Tai[Title/Abstract])) OR (Tai Chi Chuan[Title/Abstract])) OR (Taiji[Title/Abstract])) OR (Taijiquan[Title/Abstract])) OR (T'ai Chi[Title/Abstract])) OR (Tai Ji Quan[Title/Abstract])) OR (Ji Quan, Tai[Title/Abstract])) OR (Quan, Tai Ji[Title/Abstract])) OR (Baduanjin[Title/Abstract])) OR (Yijinjing[Title/Abstract])) OR (Qigong[Title/Abstract])) OR (Liuzijue[Title/Abstract])) OR (Wuqinxi[Title/Abstract])) OR (Five-animal exercises[Title/Abstract])) AND ((((((((((((((((((((((((((((((“Low Back Pain”[Mesh]) OR (Back Pain, Low[Title/Abstract])) OR (Back Pains, Low[Title/Abstract])) OR (Low Back Pains[Title/Abstract])) OR (Pain, Low Back[Title/Abstract])) OR (Pains, Low Back[Title/Abstract])) OR (Low Back Ache[Title/Abstract])) OR (Ache, Low Back[Title/Abstract])) OR (Aches, Low Back[Title/Abstract])) OR (Back Ache, Low[Title/Abstract])) OR (Back Aches, Low[Title/Abstract])) OR (Low Back Aches[Title/Abstract])) OR (Low Backache[Title/Abstract])) OR (Backache, Low[Title/Abstract])) OR (Backaches, Low[Title/Abstract])) OR (Low Backaches[Title/Abstract])) OR (Lower Back Pain[Title/Abstract])) OR (Back Pain, Lower[Title/Abstract])) OR (Back Pains, Lower[Title/Abstract])) OR (Lower Back Pains[Title/Abstract])) OR (Pain, Lower Back[Title/Abstract])) OR (Pains, Lower Back[Title/Abstract])) OR (Lumbago[Title/Abstract])) OR (Low Back Pain, Mechanical[Title/Abstract])) OR (Mechanical Low Back Pain[Title/Abstract])) OR (Low Back Pain, Posterior Compartment[Title/Abstract])) OR (Low Back Pain, Postural[Title/Abstract])) OR (Postural Low Back Pain[Title/Abstract])) OR (Low Back Pain, Recurrent[Title/Abstract])) OR (Recurrent Low Back Pain[Title/Abstract]))
Embase	1-----’traditional chinese exercises' OR ‘tai chi chuan’/exp. OR ‘tai chi chuan’ OR ‘tai ji’/exp. OR ‘tai ji’ OR ‘taiji quan’/exp. OR ‘taiji quan’ OR ‘taijiquan’/exp. OR ‘taijiquan’ OR ‘tai chi’/exp. OR ‘tai chi’ OR ‘baduanjin’/exp. OR ‘baduanjin’ OR ‘yijinjing’/exp. OR ‘yijinjing’ OR ‘qigong’/exp. OR ‘qigong’ OR ‘liuzijue’ OR ‘wuqinxi’/exp. OR ‘wuqinxi’ OR ‘five-animal exercises' 2-------’acute low back pain’ OR ‘back pain, low’ OR ‘chronic low back pain’ OR ‘loin pain’ OR ‘low backache’ OR ‘low backpain’ OR ‘lowback pain’ OR ‘lower back pain’ OR ‘lumbago’ OR ‘lumbal pain’ OR ‘lumbal syndrome’ OR ‘lumbalgesia’ OR ‘lumbalgia’ OR ‘lumbar pain’ OR ‘lumbar spine syndrome’ OR ‘lumbodynia’ OR ‘lumbosacral pain’ OR ‘lumbosacral root syndrome’ OR ‘lumbosacroiliac strain’ OR ‘pain, low back’ OR ‘pain, lumbosacral’ OR ‘strain, lumbosacroiliac’ OR ‘low back pain’
Web of Science	TS = (traditional Chinese exercises OR Tai Ji OR Tai-ji OR Tai Chi OR Tai Chi Chuan OR Taiji OR Taijiquan OR T'ai Chi OR Tai Ji Quan OR Baduanjin OR Yijinjing OR Qigong OR Liuzijue OR Wuqinxi OR Five-animal exercises) TS = (Low Back Pain OR Low Back Pains OR Low Back Ache OR Low Back Aches OR Low Backache OR Low Backaches OR Lower Back Pain OR Lumbago OR Mechanical Low Back Pain OR Postural Low Back Pain OR Recurrent Low Back Pain)
Cochrane	(traditional Chinese exercises):ti,ab,kw OR (Tai Ji):ti,ab,kw OR (Tai-ji):ti,ab,kw OR (Tai Chi):ti,ab,kw OR (Chi, Tai):ti,ab,kw OR (Tai Chi Chuan):ti,ab,kw OR (Taiji):ti,ab,kw OR (Taijiquan):ti,ab,kw OR (T'ai Chi):ti,ab,kw OR (Tai Ji Quan):ti,ab,kw OR (Ji Quan, Tai):ti,ab,kw OR (Quan, Tai Ji):ti,ab,kw OR (Baduanjin):ti,ab,kw OR (Yijinjing):ti,ab,kw OR (Qigong):ti,ab,kw OR (Liuzijue):ti,ab,kw OR (Wuqinxi):ti,ab,kw OR (Five-animal exercises):ti,ab,kw (“Low Back Pain”[Mesh]):ti,ab,kw OR (Back Pain, Low):ti,ab,kw OR (Back Pains, Low):ti,ab,kw OR (Low Back Pains):ti,ab,kw OR (Pain, Low Back):ti,ab,kw OR (Pains, Low Back):ti,ab,kw OR (Low Back Ache):ti,ab,kw OR (Ache, Low Back):ti,ab,kw OR (Aches, Low Back):ti,ab,kw OR (Back Ache, Low):ti,ab,kw OR (Back Aches, Low):ti,ab,kw OR (Low Back Aches):ti,ab,kw OR (Low Backache):ti,ab,kw OR (Backache, Low):ti,ab,kw OR (Backaches, Low):ti,ab,kw OR (Low Backaches):ti,ab,kw OR (Lower Back Pain):ti,ab,kw OR (Back Pain, Lower):ti,ab,kw OR (Back Pains, Lower):ti,ab,kw OR (Lower Back Pains):ti,ab,kw OR (Pain, Lower Back):ti,ab,kw OR (Pains, Lower Back):ti,ab,kw OR (Lumbago):ti,ab,kw OR (Low Back Pain, Mechanical):ti,ab,kw OR (Mechanical Low Back Pain):ti,ab,kw OR (Low Back Pain, Posterior Compartment):ti,ab,kw OR (Low Back Pain, Postural):ti,ab,kw OR (Postural Low Back Pain):ti,ab,kw OR (Low Back Pain, Recurrent):ti,ab,kw OR (Recurrent Low Back Pain):ti,ab,kw
China National Knowledge Infrastructure	(Title or Keywords: (“中国传统运动” OR “太极” OR “太极拳” OR “八段锦” OR “五禽戏” OR “气功” OR “六字诀”))AND(Title or Keywords: (“下腰痛” OR “下背痛” OR “慢性非特异性下腰痛” OR “慢性下腰痛” OR “非特异性下腰痛”))
Wanfang Data Knowledge Service Platform	(主题: (中国传统运动 OR 太极 OR 太极拳 OR 八段锦 OR 五禽戏 OR 气功)) AND (主题: (下腰痛 OR 下背痛 OR 慢性非特异性下腰痛 OR 慢性下腰痛 OR 非特异性下腰痛))
Chinese Science and Technology Periodical Databases	(题名或关键词: (中国传统运动 + 太极 + 太极拳 + 八段锦 + 五禽戏 + 气功 + 六字诀)) AND (题名或关键词: (下腰痛 + 下背痛 + 慢性非特异性下腰痛 + 慢性下腰痛 + 非特异性下腰痛))

## Data Availability

Data will be made available on request.

## Appendix A Appendix 1

Search strategy

## References

[bb0005] Blödt S., Pach D., Kaster T., Lüdtke R., Icke K., Reisshauer A., Witt C.M. (2015). Qigong versus exercise therapy for chronic low back pain in adults--a randomized controlled non-inferiority trial. Eur. J. Pain.

[bb0010] Bonakdar R., Palanker D., Sweeney M.M. (2019). Analysis of state insurance coverage for nonpharmacologic treatment of low back pain as recommended by the American College of Physicians Guidelines. Glob Adv Health Med..

[bb0015] Bontrup C., Taylor W.R., Fliesser M., Visscher R., Green T., Wippert P.M., Zemp R. (2019). Low back pain and its relationship with sitting behaviour among sedentary office workers. Appl. Ergon..

[bb0020] Buchbinder R., van Tulder M., Öberg B., Costa L.M., Woolf A., Schoene Mark, Croft Peter (2018). Low back pain: a call for action. Lancet.

[bb0025] Budhrani-Shani P., Berry D.L., Arcari P., Langevin H., Wayne P.M. (2016). Mind-body exercises for nurses with chronic low back pain: an evidence-based review. Nurs. Res. Pract..

[bb0030] Chen Z., Long D.Y., Wang J.Q., Chen X.Q., Xu Q., Li Y.P., Gan W., Wang F.C. (2023). Intervention study of Tai Chi in hot spring water on pain and sleep quality in elderly patients with chronic non-specific lower back pain. Jilin Medical Journal.

[bb0035] Cheng F.K. (2015). Effects of Baduanjin on mental health: a comprehensive review. J. Bodyw. Mov. Ther..

[bb0040] Ding Y., Wang J.Y. (2014). The efficacy of vertical baduanjin on middle-aged and elderly patients with chronic lower back pain. Chin. J. Gerontol..

[bb0045] Dionne C.E., Dunn K.M., Croft P.R., Nachemson A.L., Buchbinder R., Walker B.F., Wyatt M., Cassidy J.D., Rossignol M., Leboeuf-Yde C., Hartvigsen J., Leino-Arjas P., Latza U., Reis S., Real M.T.G.D., Kovacs F.M., Oberg B., Cedraschi C., Bouter L.M., Koes B.W., Picavet H.S.J., Tulder M.W., Burton K., Foster N.E., Macfarlane G.J., Thomas E., Underwood M., Waddell G., Shekelle P., Volinn E., Korff M.V. (2008). A consensus approach toward the standardization of back pain definitions for use in prevalence studies. Spine (Phila Pa 1976).

[bb0050] Du M., Hou X., Lu S., Kang T., Li Y., Wang R. (2023). Effectiveness of traditional Chinese exercise in patients with fibromyalgia syndrome: a systematic review and meta-analysis of randomized clinical trials. Int. J. Rheum. Dis..

[bb0055] Enthoven W.T., Roelofs P.D., Deyo R.A., van Tulder M.W., Koes B.W. (2016). Non-steroidal anti-inflammatory drugs for chronic low back pain. Cochrane Database Syst. Rev..

[bb0060] Essman M., Lin C.Y. (2022). The role of exercise in treating low back pain. Curr. Sports Med. Rep..

[bb0065] Ezzatvar Y., Dueñas L., Balasch-Bernat M., Lluch-Girbés E., Rossettini G. (2024). Which portion of physiotherapy treatments’ effect is not attributable to the specific effects in people with musculoskeletal pain? A meta-analysis of randomized placebo-controlled trials. J. Orthop. Sports Phys. Ther..

[bb0070] Fang L., Yan J.T., Cao Y.J., Zhang G.Y. (2015). The effect of Wuqinxi Exercise on mechanics characteristic of abdominal and back muscles and pain in patients with chronic nonspecific low back pain. Shanghai Journal of Traditional Chinese Medicine.

[bb0075] Fuentes C.J.P., Armijo-Olivo S., Magee D.J., Gross D.P. (2011). Effects of exercise therapy on endogenous pain-relieving peptides in musculoskeletal pain: a systematic review. Clin. J. Pain.

[bb0080] Furlan A.D., Yazdi F., Tsertsvadze A., Gross A., Tulder M.V., Santaguida L., Gagnier J., Ammendolia C., Dryden T., Doucette S., Skidmore B., Daniel R., Ostermann T., Tsouros S. (2012). A systematic review and meta-analysis of efficacy, cost-effectiveness, and safety of selected complementary and alternative medicine for neck and low-back pain. Evid. Based Complement. Alternat. Med..

[bb0085] Guo Y., Shi H., Yu D., Qiu P. (2016). Health benefits of traditional Chinese sports and physical activity for older adults: a systematic review of evidence. J. Sport Health Sci..

[bb0090] Hall A.M., Maher C.G., Lam P., Ferreira M., Latimer J. (2011). Tai chi exercise for treatment of pain and disability in people with persistent low back pain: a randomized controlled trial. Arthritis Care Res..

[bb0095] Huston P., McFarlane B. (2016). Health benefits of tai chi: what is the evidence?. Can. Fam. Physician.

[bb0100] Jacobs W.C., Rubinstein S.M., Willems P.C., Moojen W.A., Pellisé F., Oner C.F., Peul W.C., van Tulder M.W. (2013). The evidence on surgical interventions for low back disorders, an overview of systematic reviews. Eur. Spine J..

[bb0105] Lan C., Chen S.Y., Lai J.S., Wong A.M. (2013). Tai chi chuan in medicine and health promotion. Evid. Based Complement. Alternat. Med..

[bb0110] Li J., Bai J.Y., Li J., Shen Y.Q., Qi S.W., Feng H.L. (2022). Application of Baduanjin qigong exercise for community-based group of elderly individuals with chronic nonspecific Iow back pain. Chinese Journal of Integrative Nursing.

[bb0115] Liu J., Yeung A., Xiao T., Tian X., Kong Z., Zou L., Wang X. (2019). Chen-style tai chi for individuals (aged 50 years old or above) with chronic non-specific low back pain: a randomized controlled trial. Int. J. Environ. Res. Public Health.

[bb0120] Liu N.N., Liu H.X., Bai J.Y., Qi S.W., Feng H.L. (2021). Clinical study of baduanjin in the treatment of chronic non-specific low back pain in the community. Medicine and Hygiene.

[bb0125] Maselli F., Rossettini G., Viceconti A., Testa M. (2019). Importance of screening in physical therapy: vertebral fracture of thoracolumbar junction in a recreational runner. BMJ Case Rep..

[bb0130] Qin J.W., Zhang Y., Wu L.J., He Z.X., Huang J., Tao J., Chen L.D. (2019). Effect of tai chi alone or as additional therapy on low back pain: systematic review and meta-analysis of randomized controlled trials. Medicine.

[bb0135] Qiu B., Wang W., Tang G., Chai S., Zhang X., Zhou P., Ou Z. (2024). Long-and short-term effectiveness of traditional Chinese exercises in improving the overall physical capacity of patients with knee osteoarthritis: a systematic review and meta-analysis. Medicine.

[bb0140] Rossettini G., Emadi A.M., Dalla N.F., Testa M., Tinazzi M., Fiorio M. (2018). The placebo effect in the motor domain is differently modulated by the external and internal focus of attention. Sci. Rep..

[bb0145] Rossettini G., Campaci F., Bialosky J., Huysmans E., Vase L., Carlino E. (2023). The biology of placebo and nocebo effects on experimental and chronic pain: state of the art. J. Clin. Med..

[bb0150] Santos C., Donoso R., Ganga M., Eugenin O., Lira F., Santelices J.P. (2020). Low back pain: Review and evidence of treatment. Medical journal clinical Las Condes.

[bb0155] Schiavo J.H. (2019). PROSPERO: an international register of systematic review protocols. Med. Ref. Serv. Q..

[bb0160] Sherman K.J., Cherkin D.C., Connelly M.T., Erro J., Savetsky J.B., Davis R.B., Eisenberg D.M. (2004). Complementary and alternative medical therapies for chronic low back pain: what treatments are patients willing to try?. BMC Complement. Altern. Med..

[bb0165] Stochkendahl M.J., Kjaer P., Hartvigsen J., Kongsted A., Aaboe J., Andersen M., Andersen M., Fournier G., Højgaard B., Jensen M.B., Jensen L.D., Karbo T., Kirkeskov L., Melbye M., Morsel-Carlsen L., Nordsteen J., Palsson T.S., Rasti Z., Silbye P.F., Steiness M.Z., Simon T., Vaagholt M. (2018). National Clinical Guidelines for non-surgical treatment of patients with recent onset low back pain or lumbar radiculopathy. Eur. Spine J..

[bb0170] Sun D., Wang R., Sun D., Chen J. (2021). Clinical study on baduanjin exercise in sitting for low back pain induced by osteoporosis in senile male patients. Journal of New Chinese Medicine.

[bb0175] Teut M., Knilli J., Daus D., Roll S., Witt C.M. (2016). Qigong or yoga versus no intervention in older adults with chronic low back pain-a randomized controlled trial. J. Pain.

[bb0180] Tugwell P., Tovey D. (2021). PRISMA 2020. J. Clin. Epidemiol..

[bb0185] Wang X., Zhu Q.B., Fang F.F., Gu W. (2017). Clinical observation of Baduanjin in treating chronic lumbago male patients in the elderly. Chinese Journal of Traditional Chinese Medicine.

[bb0190] Wang X., Song W.J., Ruan Y., Li B.C., Lü C., Huang N., Fang F.F., Gu W. (2022). Core muscle functional strength training for reducing the risk of low back pain in military recruits: an open-label randomized controlled trial. J. Integr. Med..

[bb0195] Wang F., Cai J., Liu J., Duan B., Yang Y., Yang Q. (2023). Effects of traditional Chinese exercise on physiological indicators and quality of life in patients with coronary heart disease: a systematic review and meta-analysis. Medicine.

[bb0200] Xiao L., Duan H., Li P., Wu W., Shan C., Liu X. (2020). A systematic review and meta-analysis of Liuzijue in stable patients with chronic obstructive pulmonary disease. BMC Complement Med Ther..

[bb0205] Xie J., Guo J., Wang B. (2024). Comparing the effectiveness of five traditional Chinese exercises in improving balance function in older adults: a systematic review and Bayesian network meta-analysis. PeerJ.

[bb0210] Yan Z.W., Yang Z., Yang J., Chen Y.F., Zhang X.B., Song C.L. (2022). Tai chi for spatiotemporal gait features and dynamic balancing capacity in elderly female patients with non-specific low back pain: a six-week randomized controlled trial. J. Back Musculoskelet. Rehabil..

[bb0215] Yang B., Li H., Zhang T., He X., Xu S. (2012). The incidence of adjacent segment degeneration after cervical disc arthroplasty (CDA): a meta analysis of randomized controlled trials. PLoS One.

[bb0220] Yang D., Huang H., Xu D.D., Zhao Y. (2013). Effects of Baduanjin exercise on patients with chronic nonspecific low back pain and surface electromyography signs of erector spinal muscle: a randomized controlled trial. Medicine.

[bb0225] Ye L., Jiang C. (2021). Reflections on the related values of five animals-exercises from the perspective of national culture. International Journal of Social Science and Education Research..

[bb0230] Yuan Q.L., Guo T.M., Liu L., Sun F., Zhang Y.G. (2015). Traditional Chinese medicine for neck pain and low back pain: a systematic review and meta-analysis. PLoS One.

[bb0235] Zheng W., Li M., Hong Y., Xie F., Yan Q., Peng Z., Huang H., Liao H., Liu X. (2019). Traditional Chinese exercise (TCE) on pulmonary rehabilitation in patients with stable chronic obstructive pulmonary disease: protocol for a systematic review and network meta-analysis. Medicine.

[bb0240] Zhu G.F., Shen Z.F., Shen Q.H., Jin Y.Q., Lou Z.Y. (2017). Effect of Yi Jin Jing (sinew-transforming qigong exercises) on skeletal muscle strength in the elderly. J. Acupunct. Tuina Sci..

[bb0245] Zou L., Zhang Y., Liu Y., Tian X., Xiao T., Liu X., Yeung A.S., Liu J., Wang X., Yang Q. (2019). The effects of tai chi Chuan versus core stability training on lower-limb neuromuscular function in aging individuals with non-specific chronic lower back pain. Medicina.

